# Fresh, Mechanical, and Thermal Properties of Cement Composites Containing Recycled Foam Concrete as Partial Replacement of Cement and Fine Aggregate

**DOI:** 10.3390/ma16227169

**Published:** 2023-11-15

**Authors:** Jan Pizoń

**Affiliations:** Faculty of Civil Engineering, Silesian University of Technology, 44-100 Gliwice, Poland; jan.pizon@polsl.pl

**Keywords:** foam concrete, cement, thermal properties, recycled concrete aggregate, sustainable building materials, lightweight aggregate

## Abstract

The research presented in this article was conducted to evaluate the suitability of recycled foam concrete (RFC) as an ingredient in newly created cement mortars. The basis for an analysis was the assumption that the waste is collected selectively after separation from other waste generated during demolition. The motivation for the research and its main problem is a comparison of the performance of RFC used in various forms. RFC was used in two forms: (1) recycled foam concrete dust (RFCD) as a 25 and 50% replacement of cement, and (2) recycled foam concrete fine aggregate (RFCA) as a 10, 20, and 30% replacement of sand. The basic properties of fresh and hardened mortars were determined: consistency, density, initial setting time, absorbability, compressive strength, thermal conductivity coefficient, and heat capacity. Research is complemented with SEM observations. The properties of fresh mortars and mechanical parameters were decreased with the usage of any dosage of RFC in any form, but the thermal properties were improved. The required superplasticizer amount for proper consistency was raised four times for replacing cement with 50% of RFCD than for 25% of such replacement. The mix density dropped by about 8% and 9% for mortars with the replacement of 50% cement by RFCD and 30% sand by RFCA in comparison to reference mortar. A 30% decrease in initial setting time was observed for cement replacement. In the case of sand replacement, it was the opposite—an increase of 100%. The dry density decreased by about 14% and 11% for mortars with the replacement of 50% cement by RFCD and 30% sand by RFCA in comparison to reference mortar. Absorbability was raised by about two times after replacement with both RFCD and RFCA. Compressive strength after 28 days dropped significantly by 75% and 60%, and the thermal conductivity coefficient decreased by 20% and 50% with 50% RFCD added instead of cement and 30% RFCA replacing sand. It indicates greater efficiency in thermomechanical means from RFCA in comparison to RFCD. This material can be used especially in the production of plaster and masonry mortar. Linear correlations of dry density and thermal conductivity coefficient and the latter and compressive strength were proven as reliable for RFCD replacement of cement and RFCA replacement of sand in mortars with greater w/c ratio.

## 1. Introduction

The construction industry generated 37% of process- and energy-related CO_2_ emissions and more than 34% of global energy demand in 2021. The latter percentage increased by 4%pt. compared to the previous year. In Europe, the construction industry caused 40% of CO_2_ emissions, about 80% of which came from burning fossil fuels [1]. The cement industry is responsible for a large percentage of these emissions. Each ton of cement produced is 0.6 tons of carbon dioxide emitted [2]. On an annual basis, this is about 8% of global CO_2_ emissions [3].

One of the possible paths that can be followed to reduce these values is the usage of waste materials. This could be industrial waste, which has been the subject of other publications by the author [4,5,6], dust and ash from municipal waste incineration [7,8], plastics [9,10], used tires [11,12], glass [11,13,14], or waste from demolition of existing structures [11,15,16], and many others [17,18,19]. Waste materials can be used as substitutes for natural aggregate or cement.

In this article, the author focused on the waste generated by the demolition of the insulation layers of an existing building. The material was used in the form of fine aggregate (recycled foam concrete aggregate—RFCA) as a substitute for sand, and dust was used as a substitute for cement (recycled foam concrete dust—RFCD). The reuse of this type of construction demolition waste (CDW) in newly constructed buildings fits into the global idea of sustainable development and circular economy. It also fits in with the idea of reducing energy consumption for heating and cooling of habitable spaces.

Foam concrete is a material formed by mixing cement paste with a foaming agent [20]. It may contain sand in its composition in limited amounts, but as a general rule, it does not contain coarse aggregate. Foam concrete can also be supplemented with lightweight aggregates such as expanded vermiculite or perlite [21]. The foam concrete used in this study did not contain any aggregate. There are also studies on the use of waste materials to produce new foam concrete [21,22]. It is a lightweight material with a density of 300–1800 kg/m^3^ [23,24]. The main property of foam concrete is its low heat transfer coefficient. Its value depends primarily on density but also on moisture content and the presence of additional materials used in its production [25]. Sources [25,26] report heat transfer coefficient values of 0.1 to 0.7 and 0.389 to 0.734 Wm^−1^ K^−1^ for densities of 600 to 1600 kg/m^3^. Thus, after crushing foam concrete, lightweight aggregate can be obtained, and after grinding it, dust consisting of hydrated cement can be obtained. The compressive strength of foam concrete decreases proportionally to decreasing density. This is an exponential relationship. Compressive strength values according to [27,28,29,30,31] range from <1 MPa for foam concrete with a density of 400 kg/m^3^ through 3–5 MPa for foam concrete with a density of 800 kg/m^3^, 10–12 MPa for foam concrete with a density of 1200 kg/m^3^ to 15–20 MPa for foam concrete with a density of 1400 kg/m^3^.

Foam concrete waste can be used as a lightweight aggregate. Common lightweight aggregates used for lightweight concrete are expanded perlite [32,33,34], vermiculite [34,35], expanded clay [36], pumice [37,38], and granulated polystyrene [34,35]. Waste materials—glass, sludge, slag, etc.—can also be used as lightweight aggregate [39,40]. There are few reports on the use of such waste materials as an ingredient in new cement composites [41]. A problem that can be encountered with the use of lightweight aggregates is their high water absorption and the associated problem of achieving adequate workability of the mix [42,43,44]. However, it can be a method of internal concrete curing, which is an advantage [43].

There are many studies on the use of waste materials as an ingredient in new lightweight aggregate concretes. Materials used include recycled expanded polystyrene (EPS), polyurethane waste, and agricultural waste. Recycled Autoclaved Aerated Concrete (AAC) and Cellular Lightweight Concrete (CLC) are also known to be used. Some of these are referred to below.

Based on the publication [45], recycled expanded polystyrene (EPS) can be used to partially or fully replace natural aggregate. In this case, even an improvement in consistency was noted compared to mortars containing only natural sand. Thermal insulating properties were also improved, while mechanical performance deteriorated significantly.

The authors [46] report the possibility of using coarse rigid polyurethane foam waste as a component of new lightweight concretes. A decrease in mechanical properties, in comparison to normal-weight concrete, was observed. However, it almost satisfied the criteria of structural lightweight aggregate.

The publication [47] provides an overview of agricultural waste materials as potential components of lightweight concrete. These include oil palm shells, coconut shells, corn cobs, cork waste, and waste plastic. In some cases, improvements in mechanical properties were shown but probably were related to a reduction in the effective water–cement ratio. A problem with maintaining the appropriate consistency was also indicated, but also the beneficial aspect of the internal self-curing of the concrete. The durability challenge and the possible segregation problems were also pointed out.

The article [48] deals with the use of crushed cellular concrete as an aggregate for new cement composites. A decrease in compressive strength and density and a deterioration in consistency while improving the thermal conductivity coefficient were shown. The decrease in compressive strength was reported as 32% up to 72% for the mix, with 20% up to 100% replacement of natural aggregate with lightweight one. The thermal conductivity coefficient was 2.07 (W/mK) for the reference sample and dropped down to 1.18 (W/mK) for 100% substitution of both natural coarse and fine aggregates.

Other types of lightweight demolition waste are Autoclaved Aerated Concrete (AAC) and Cellular Lightweight Concrete (CLC). The effect of these wastes, when used as an aggregate, on the properties of new mortars is described in the article [49]. The use of both types of aggregate reduced the compressive strength from about 24 MPa for mortars containing only natural sand to about 9 MPa and 12 MPa for the replacement of sand with CLC and AAC at 30%, respectively.

AAC as a lightweight aggregate was also the subject of an article [50]. Possibilities of AAC waste usage as concrete aggregate, prefabricated concrete tiles, concrete blocks, and others were proven for non-load-bearing purposes.

The article [51] also provides research results with the use of AAC as an aggregate for lightweight concrete. The strength results when aggregate was fully substituted with AAC was in the range of 2.5–16 MPa, depending on the mix composition. Thermal conductivity coefficient ranged from 0.28 to 0.45 (W/mK).

There are fewer reports on the use of powdered lightweight concrete as a replacement for cement. There are publications describing AAC concretes, but their production does not include a foaming agent, which can affect the properties of the concrete. The dust obtained by grinding foam concrete can be used as a replacement for cement or as an almost inert filler. This is much more popular. It is reported that with small amounts of dust used in this way, it is possible to obtain undeteriorated or even enhanced properties of the mixture and hardened concrete. One must consider the decreased consistency and the need to use water-reducing admixtures [52,53,54,55,56].

The article [50] describes the possibility of replacing part of the cement with AAC waste, which improved the freeze–thaw resistance and other properties of the new composite.

The effect of AAC and CLC waste, used as a cement replacement, on the properties of new mortars is described in the article [49]. When the cement was replaced with powdered AAC, the strength decreased regardless of the amount of waste added—from about 24 MPa for the reference mortar to about 18 MPa for the mortar with a 30% cement replacement. In the case of ground CLC for 5–20% cement substitution, an improvement in mechanical properties was noted. It was an increase from approx. 24 MPa to approx. 28 MPa for 10% cement substitution.

**Justification for addressing the topic**

The research presented in this article was conducted to evaluate the suitability of specific demolition waste for reuse as an ingredient in cement mortars. Prospective uses for the composites so created could include traditional mortars as well as mortars with insulating properties for floor layers, roofs, or masonry mortars. A waste material from the demolition of an insulation layer made of foam concrete was used. The basis for a credible analysis was the assumption that this waste is collected selectively after separation from other waste generated during demolition. A particularly valuable aspect is the common determination of thermal and strength properties. The RFC was used in two different forms—dust (RFCD), which is a replacement for cement, and a fine aggregate of size 0–4 mm (RFCA). The usage of recycled foam concrete as a part of fine aggregate is present in many publications. However, the use of this material as a replacement for cement is much less well recognised. In this paper, the latter is also included, described, and compared with the former. According to the statements above, it is worthwhile to compare the possibility and amount of waste used if it is treated as a substitute for sand or cement. This article will partially fill the gap in publications on the use of waste foam concrete as a fine aggregate for cement composites.

## 2. Materials and Methods

### 2.1. Materials

Cement mortars were prepared to assess the influence of RFC on the properties of cement composites. RFC was used in two forms: (1) recycled foam concrete dust (RFCD) with particles up to 0.063 mm or 0.125 mm, and (2) recycled foam concrete fine aggregate (RFCA) with particles up to 4 mm. Three series of samples were produced: (1) RFCD replacing 25% and 50% of cement, (2) RFCA replacing 10% and 20% of natural sand at water–cement ratio 0.5, and (3) RFCA replacing 10%, 20%, and 30% of natural sand at water–cement ratio 0.6 (denoted as (W+) symbol).

As a binder, cement or cement-RFCD mixtures were used. Cement CEM I 42.5R was obtained from one of Polish cement manufacturers as a fine aggregate for mortars natural sand (NS) and recycled foam concrete aggregate (RFCA). CEN standard sand and tap water were used. PCE-based superplasticizer (SP) was used to maintain the same consistency for all mixes. SP was added regarding total binder mass, not exceeding 5% of it. Exact composition of mortars is given in Table 1.

**Figure 1:**
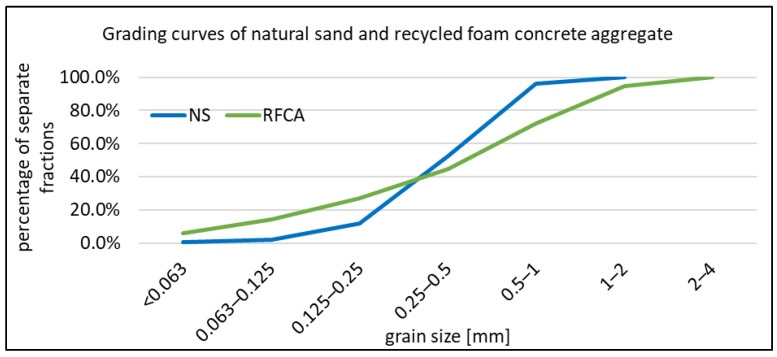
Grading curve of NS and RFCA.

RFC, which originated from an existing structure, was obtained from a demolition company. It played the role of insulation of flat roof structure. RFCA was crushed into 0–4 mm grade. In this form, it was used as a 10–30% replacement for natural sand. It is impossible to use more RFCA because of the consistency drop. After crushing, it was ground to obtain 0.0–0.250 mm and sieved to separate RCFD fractions of 0.0–0.063 mm and 0.0–0.125 mm, respectively. The former was used as a 25% replacement of cement. The latter was used as 25% and 50% replacement of cement. Bulk densities and grading of RFCA and NS are presented in Table 2. The grading curve of NS and RFCA is given also in Figure 1.

### 2.2. Methods

Properties of mortars were tested according to European standards:

- Initial setting time: EN 480-2:2008 Admixtures for concrete, mortar, and grout—Test methods—Part 2: Determination of setting time [60];
- Consistency: EN 1015-3:2000/A2:2007 Methods of test for mortar for masonry—part 3: determination of consistence of fresh mortar (by flow table) [59];
- Density of fresh mortar: EN 1015-6 Methods of test for mortar for masonry—part 6: Determination of bulk density of fresh mortar [61];
- Compressive strength: EN 196-1 Methods of testing cement—Part 1: Determination of strength [58];
- Density of hardened mortar: EN 1015-10 Methods of test for mortar for masonry—part 10: Determination of dry bulk density [62];
- Absorbability was tested on samples prepared for density test, cured in water for 28 days, weighted, and dried to constant mass;
- Microstructure observations: The examination was performed using an electron scanning microscope (JEOL JSM-7200F; JEOL company, Tokyo, Japan) equipped with an EDS analyzer;
- Thermal properties were assessed with ISOMET 2114 Portable Thermal Properties Analyzer (Applied Precision s.r.o., Bratislava, Slovakia). Samples used for these tests were cubic 10 × 10 × 10 cm. Determination of thermal properties using the ISOMET instrument is based on the ‘hot plate’ method and involves analysing changes in the surface temperature of the test sample in two phases. Firstly, while it is being heated at constant power, and secondly, while it is being cooled. Thermal conductivity (λ) $\left[\frac{W}{m\cdot K}\right]$ and volume heat capacity (cρ) $\left[\frac{J}{m^{3}\cdot K}\right]$ were recorded.

## 3. Results

### 3.1. Fresh Mix Properties

#### 3.1.1. Consistency

**Figure 2:**
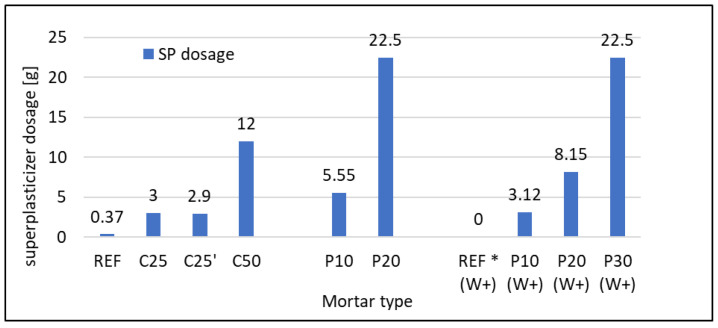
Consistency represented as a superplasticizer amount necessary to obtain constant flow diameter of mortars. * without superplasticizer added, the reference mortar of w/c = 0.6 reached more plastic con-sistency than required.

**Figure 3:**
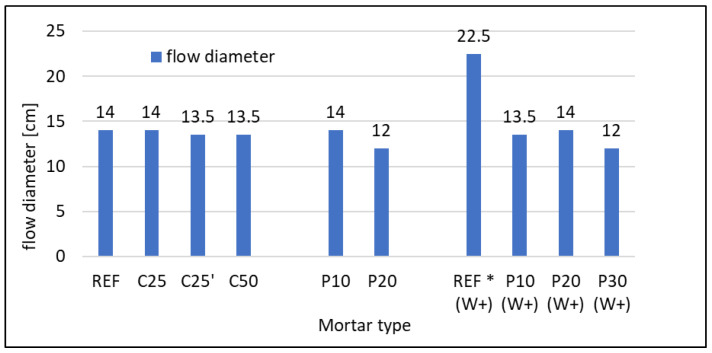
Flow diameter of mortars. * without superplasticizer added, the reference mortar of w/c = 0.6 reached more plastic con-sistency than required.

Because of the significant decrease in consistency of mortars and, thus, the impossibility of conducting measurements for mortars containing both RFCD and RFCA, it was essential to use different methods to assess it. The superplasticizer (SP) was added in the proper amount to obtain the same or similar (13 ± 1 cm) diameter of flow. The amount of SP added collated with flow diameter is presented in Figure 2 and Figure 3, respectively. It was predictable that in both cases of RFC usage, it would enhance the water demand and, thus, decrease the consistency. The necessary SP amount raised from 0.37 g up to 2.9 and 3.0 g for 25% cement replacement by RFCD, but it raised greatly (up to 12 g) for 50% of such replacement. In the first case, the behaviour of cement mixed with RFCD ground to 0.063 mm and 0.125 mm is similar, suggesting that in terms of consistency, it is not viable to grind to the finest grain size. In the second case, the use of SP in such large quantities may not be economically or environmentally justified. In the case of composites with w/c = 0.5, substituting 10% of the sand with RFCA resulted in the need for 5.55 g of SP, and increasing this percentage to 20% required as much as 22.5 g of SP—the maximum dose allowed by the standard (5% m.c.). In the case of composites with w/c = 0.6, the reference mortar did not require SP, and even so, the resulting flow diameter was much greater than that assumed at the beginning. Substituting 10% sand for RFCA necessitated the use of 3.12 g of SP, and increasing this percentage to 20% required 8.15 g of SP, and only substituting 30% required the maximum dose allowed by the standard (5% m.c. = 22.5 g).

#### 3.1.2. Fresh Density

**Figure 4:**
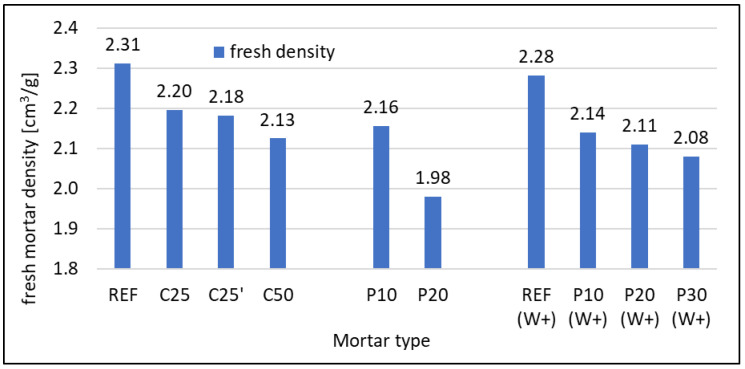
Density of mortars in plastic state.

The density of fresh mortars is given in Figure 4. The reference mortar for w/c = 0.5 had a density of 2.31 g/cm^3^. For w/c = 0.6, it was slightly lower at 2.28 g/cm^3^. With a 25% RFCD cement substitution, the density was lower but comparable regardless of the fraction of RFCD used. With 50% cement replacement, the density dropped to 2.13 g/cm^3^.

With a 10% sand substitution with RFCA, the density decreased to 2.16 and 2.14 g/cm^3^ for mortar with w/c = 0.5 and w/c = 0.6, respectively. Differences can be seen for mortars with a 20% sand replacement with RFCA, where in the case of the composite with w/c = 0.5, large amounts of SP had to be used, which could lead to excessive aeration of the mix. This resulted in a large drop in density. This effect is not seen with mortars with w/c = 0.6. With a 30% sand replacement in the latter, the density dropped to 2.08 g/cm^3^, which still places this composite in the range of normal mortars.

#### 3.1.3. Initial Setting Time

**Figure 5:**
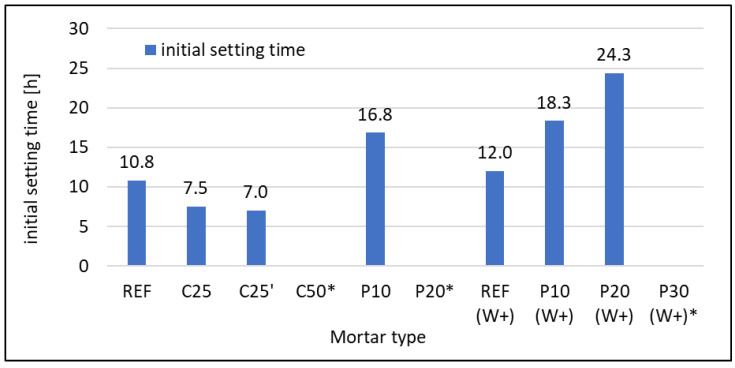
Initial setting time. * It was not possible to obtain standard consistency (acc. to EN 196-3 [57]) with max allowed admixture dosage (5% of cement mass).

Initial setting time is very important from a practical point of view. If the time is too long, the required mechanical properties will not be achieved quickly enough, and if it is too short, the workability period of the mix will be shortened. In the case of the mixtures tested, all times are within the desired range for practical reasons, even if they look too long. This is caused by the method, according to EN 480-2 [60], that requires testing using mortars, for which the initial setting time is much longer than for respective standard consistency cement paste tested according to EN 196-3 [57]. The results are given in Figure 5.

An interesting relationship is noticeable. When replacing cement with RFCD, the initial setting time decreases compared to the reference mortar, while when replacing sand with RFCA, the time increases. When using 25% RFCD, the initial setting time decreases by about 30–35%, depending on the fraction of dust used. A reference mortar with w/c = 0.6 has a longer initial setting time than one with w/c = 0.5, which is entirely normal. The same relationship applies to mortars with 10% RFCA content. In this case, the initial setting time is approximately 50 and 60% longer than for reference mortars with w/c = 0.6 and 0.5, respectively. The initial setting time for a mortar in which 20% sand has been substituted for RFCA is even longer and may be considered impractical, but there are no standards describing the maximum initial setting time. The mixes C50, P20, and P30(W+) could not be examined because of exceeding the maximum dosage of SP to obtain standard consistency. Further adding of SP may lead to significantly unreliable results; thus, mixtures were not subjected to tests. Note that admixtures that accelerate the setting can be used to modify the initial setting time [63].

### 3.2. Hardened Composites Properties

#### 3.2.1. Density

**Figure 6:**
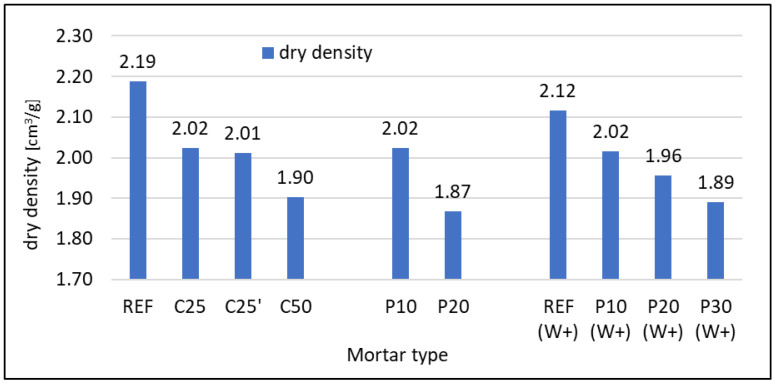
Density of oven-dried hardened mortars after 28 days of curing.

The dry density of hardened mortars is similar to that of fresh mortars. The results are given in Figure 6. As the RFCD or RFCA content increases, the density decreases. The fineness of RFCD added as a cement replacement has no effect on density. The addition of 10% RFCA results in the same reduction in density for mortars with w/c = 0.5 and 0.6. As with the density of the fresh mortar, differences can be observed in mortars with 20% RFCA, in which large amounts of SP had to be used for a composite with w/c = 0.5, which could lead to excessive aeration of the mix. This resulted in a large decrease in density. This effect is not found in mortars with w/c = 0.6 with a lower SP amount.

#### 3.2.2. Absorbability

**Figure 7:**
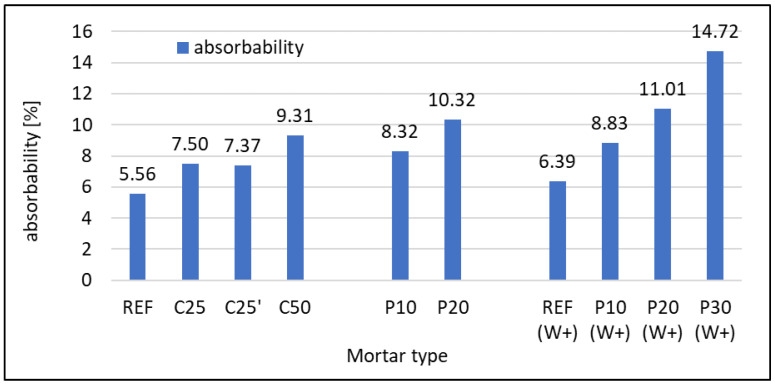
Absorbability of hardened mortars after 28 days of curing.

The absorbability of hardened mortars is ruled by an inverse relationship to density. The more recycled foam concrete material, the higher the absorbability of the mortars. The results can be seen in the graph in Figure 7. In this case, it is also observed that the fineness of RFCD as a cement replacement has no effect on absorbability. Mortars containing RFCA as a substitute for sand show higher absorbability than those containing RFCD as a substitute for part of the cement. It should be noted that, in absolute terms, the RFCD content in C25 and C50 mortar is 112.5 g and 225 g, respectively, while the RFCA content in P10 and P20 mortar is 135 g and 270 g, respectively. This gives a ratio (Cxx/Pyy) of 0.83 and, in terms of the equivalent for P10 and P20 mortars, an absorbability of 6.91% and 8.57%, respectively. These values are slightly lower but comparable to those for C25 and C50 mortars, respectively.

#### 3.2.3. Compressive Strength

**Figure 8:**
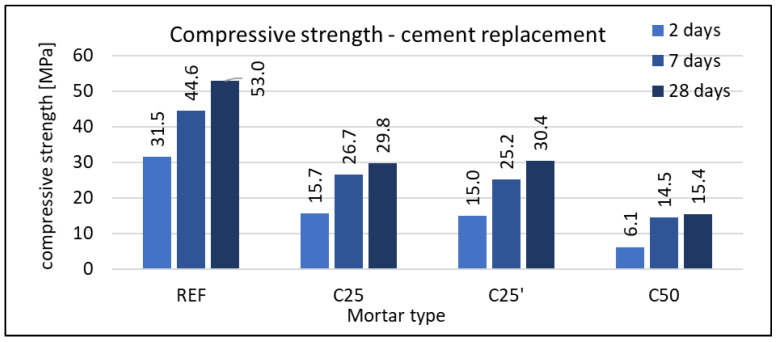
Compressive strength of mortars with RFCD replacement of cement.

**Figure 9:**
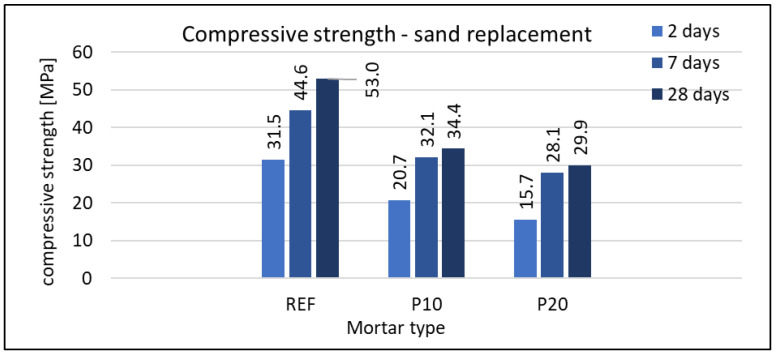
Compressive strength of mortars with w/c = 0.5 and RFCA replacement of sand.

**Figure 10:**
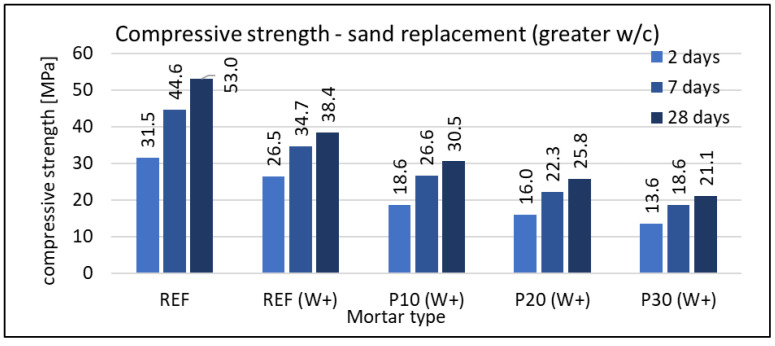
Compressive strength of mortars with w/c = 0.6 and RFCA replacement of sand.

The compressive strength was tested on mortar samples with dimensions of 160 × 40 × 40 mm. Each type of mortar, at each date (2, 7, and 28 days), was tested using six samples. In neither case was a standard deviation greater than 3% of the average value recorded. Results are given in Figure 8, Figure 9 and Figure 10.

The use of waste foam concrete as a replacement for cement reduces the strength of the composite compared to the reference mortar. This effect is evident in all terms. Detailed results are shown in Figure 8. For the mortar containing 25% RFCD, the relative strength gain between 7 and 28 days is similar to that of the reference mortar. For the mortar with 50% RFCD, no clear strength improvement is observed after the 7th day. As with other properties of hardened mortars, the maximum grain size of RFCD has no effect on compressive strength.

RFCA used as a replacement for sand also reduced the compressive strength in all terms. The graphs are shown in Figure 9 and Figure 10 for mortars with w/c ratio = 0.5 and 0.6, respectively. It is noteworthy that a much greater difference in strength was recorded between the reference mortar and that containing 10% RFCA than between the latter and that containing 20% RFCA. However, this only applies to mortars with a w/c ratio of 0.5. At w/c = 0.6, the differences are proportional to the foam concrete waste content. A very important factor influencing the mechanical properties of cement-based composites is specifically the w/c ratio. This influence is somewhat less in the case of mortars containing lightweight aggregate. This can be seen in both types of mortar (P10 and P20) compared to their counterparts with higher w/c (P10(W+) and P20(W+)). For example, for the 28-day strength, the REF/REF(W+) ratio is 1.38, while for those containing foam concrete waste, these values are lower. For P10/P10(W+), it was 1.12, and for P20/P20(W+), it was 1.15. Lightweight aggregate absorbs more water than natural aggregate and reduces the w/c ratio of the cement paste. This new ratio is called the effective water-to-cement ratio. The absorbed water will also act as a reservoir for the water needed to cure the cementitious composite and will allow internal self-curing of the mortar containing RFCA.

#### 3.2.4. Thermal Properties

**Figure 11:**
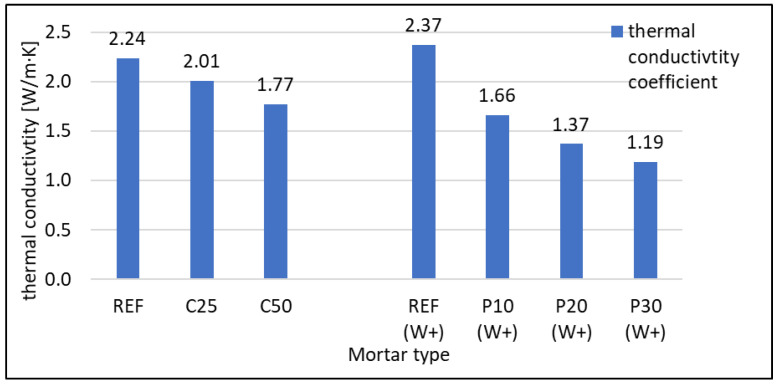
Thermal conductivity coefficient of RFCD- and RFCA-containing mortars.

**Figure 12:**
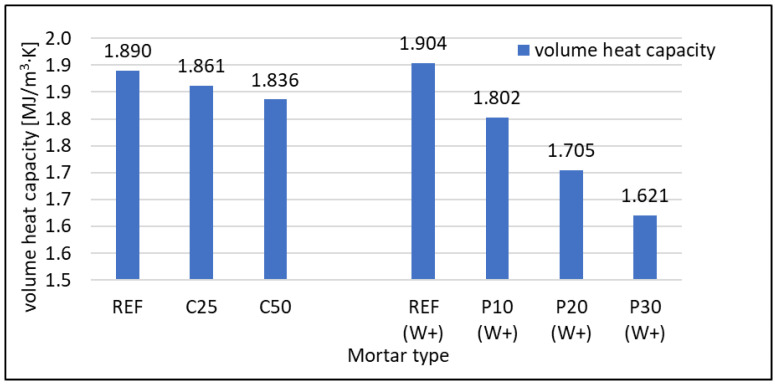
Volumetric heat capacity of RFCD- and RFCA-containing mortars.

Thermal properties were assessed with ISOMET 2114. Thermal conductivity (λ) $\left[\frac{W}{m\cdot K}\right]$ and volume heat capacity (cρ) $\left[\frac{J}{m^{3}\cdot K}\right]$ tests results are presented in Figure 11 and Figure 12, respectively. Both values decrease together with the rising content of foam concrete waste in mortar. In the case of RFCD replacing cement, λ decreased by about 10 and 20% for 25 and 50% cement replacement with waste, respectively. For RFCA replacing sand, the decrease was much more significant at 30%, 43%, and 50% for 10, 20, and 30% replacement, respectively. It should be remembered that a 20% level of sand replacement with RFCA allows more waste to be used than a 25% cement replacement with RFCD. The better insulating capacity of waste RFCA than RFCD is due to the higher air content. This results in better behaviour of mortars containing RFCA. The differences seen in heat capacity are not as evident as λ for mortars with RFCD content as a replacement for part of the cement. The ability to hold heat decreases with increasing waste content, but the difference is close to zero. When RFCA is used as a substitute for sand, the decrease is more pronounced, reaching about 15% at 30% RFCA content, a much smaller decrease than in the case of thermal conductivity.

**Figure 13:**
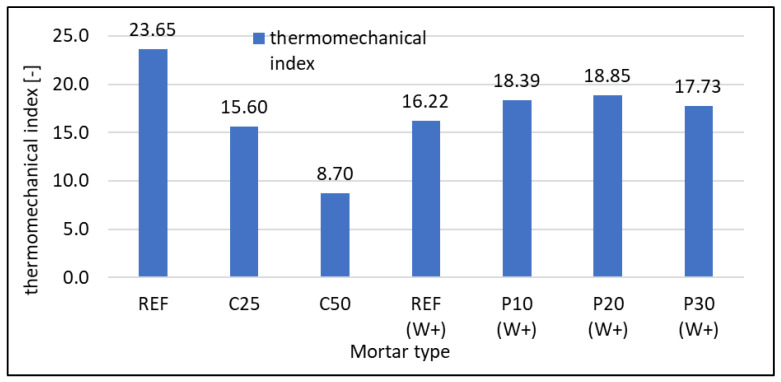
Thermomechanical index of tested mortars calculated according to formula proposed by [64].

According to Nowak et al. [64], the authors proposed a methodology for comparing concretes containing waste not in terms of mechanical properties alone but these properties in combination with the thermal conductivity coefficient. The equation φ = f_c_/λ, which connects compressive strength (f_c_) and thermal conductivity coefficient (λ) in the form of the thermomechanical index (φ), was proposed. Due to the lack of a physical connection, the authors decided not to include units for the value obtained from it. This index is of practical use, assuming that sufficiently high standards for the strength of the concrete are maintained. In this way, material selection can be optimised. The proposed solution seems reasonable due to the fact that most waste materials deteriorate mechanical properties, but some of them can improve insulating properties. Thinking about the equal importance of the mechanical performance of concrete and other characteristics makes the assessment of the material’s functionality a more complicated case. This is evident in the situation when a composition change of the concrete mix is followed by beneficial changes in some of the properties and unfavourable changes in other parameters. Figure 13 shows the values of this coefficient. For mortars containing RFCD as a replacement for cement, its value decreases proportionally to the waste content. For the replacement of sand with RFCA, the coefficient is similar to the reference sample but slightly higher for mortars containing foam concrete waste. The highest values are recorded for 10% and 20% of sand replacement. This is related to the preservation of sufficient strength and the reduction of the thermal conductivity coefficient. At RFCA content of 30%, the value of this index is lower. For practical reasons, it is not possible to prepare a mortar with a higher RFCA content than 30%, but further deterioration of the index is likely to be seen.

#### 3.2.5. Correlations of Mortars Properties

**Figure 14:**
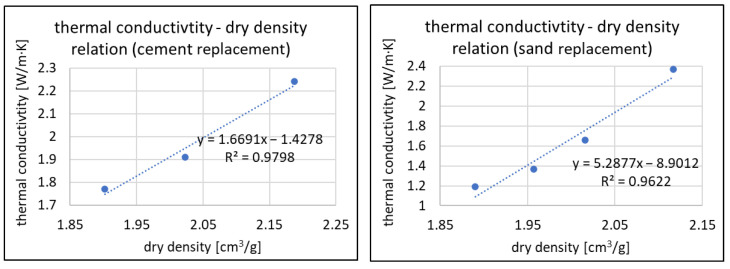
Thermal conductivity coefficient—dry density relations for RFCD (**left**) and RFCA (**right**).

**Figure 15:**
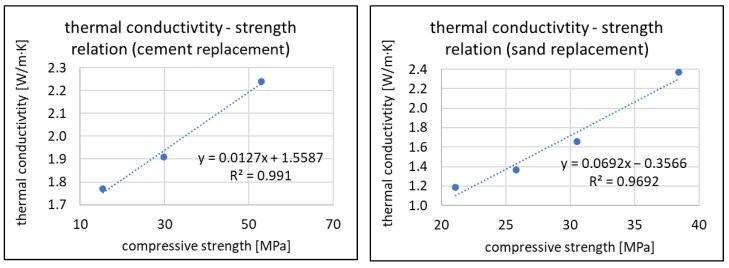
Thermal conductivity coefficient—compressive strength relations for RFCD (**left**) and RFCA (**right**).

In Figure 14 and Figure 15, the relationships of thermal conductivity coefficient to density and thermal conductivity coefficient to compressive strength are shown. The graphs show that within the assumed range of amounts of RFCD and RFCA as a substitute for cement and sand, respectively, these relationships can be approximated to linear while maintaining reasonably accurate results. This is evidenced by R^2^ ratios above 0.96.

#### 3.2.6. Microstructure

**Figure 16:**
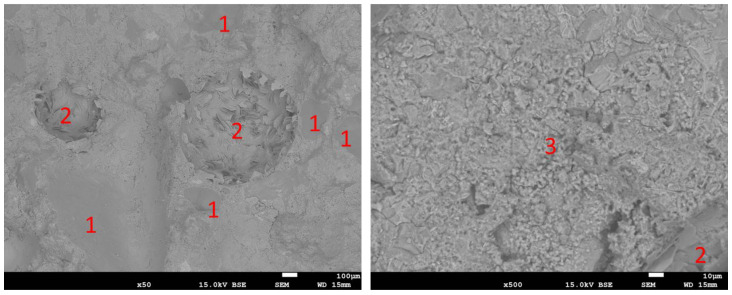
SEM images of REF sample. 1—sand particles; 2—air pores filled with portlandite; 3—CSH phase.

**Figure 17:**
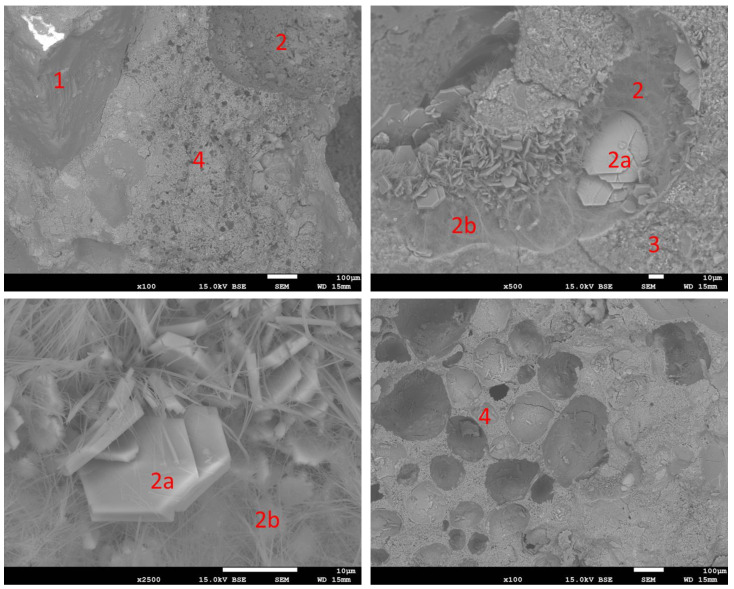
SEM images of P-20 sample. 1—sand particles; 2—air pores filled with 2a—portlandite, 2b—ettringite; 3—CSH phase; 4—particle of foam concrete.

**Figure 18:**
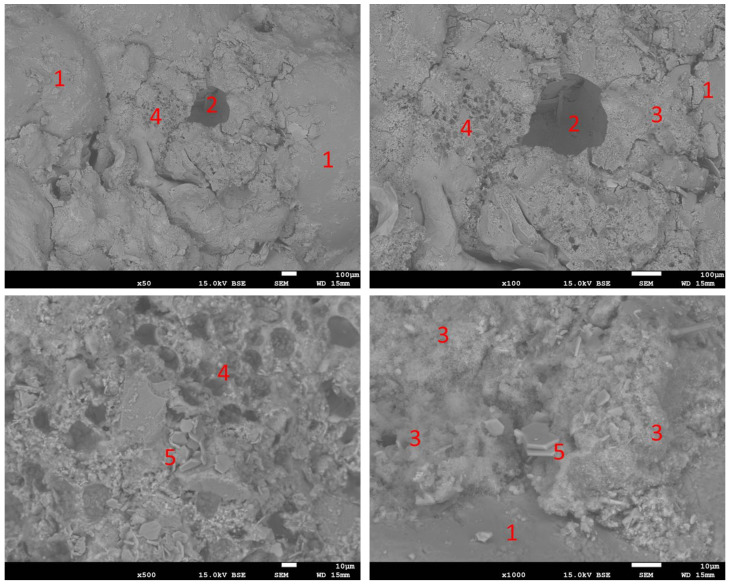
SEM images of C-50 sample. 1—sand particles; 2—air pores; 3—CSH phase; 4—particle of foam concrete; 5—portlandite.

Observations with the SEM revealed the normal microstructure of the reference sample. This is shown in Figure 16. Sand grains (1) surrounded by cement paste can be seen, in which most of the observed area is the CSH phase (3), among which air pores (2) filled with hexagonal portlandite crystals can be found. The microstructure of samples containing RFCD and RFCA foam concrete is shown in Figure 17 and Figure 18, respectively. These have the same elements, additionally complemented by foam concrete particles with a clearly visible porous structure. Also visible in the pores of these mortars are needles of ettringite next to plates of portlandite in the air pores. In the case of the sample in which the cement has been replaced with RFCD, the foam concrete particles are noticeably smaller in size than in the case of the RFCA replacing sand, which are similar in size to the sand particles.

## 4. Discussion

Deterioration of mortar consistency by substituting part of the natural aggregate with recycled concrete aggregate is normal. This is caused by the increased absorbability of the recycled aggregate [65,66]. The magnitude of the phenomenon is even greater as the aggregate used is a lightweight aggregate, being obviously more porous [67,68]. The superplasticiser used made it possible to test the properties of the fresh mortars and to prepare samples for mechanical properties tests up to 50% RFCD and 30% RFCA. Other, potentially more effective admixtures can be used to further improve consistency and the possibility of making composites containing a greater amount of waste. Further increases in the amount of water are not recommended due to the need to maintain mechanical parameters at an appropriate level.

Some SPs can lead to excessive aeration of the mix if used in overly large quantities [69]. This is evident in the density of fresh and hardened mortars.

An interesting relationship can be observed in the change in the initial setting time of mortars where cement or sand is substituted. When RFCD is used instead of part of the cement, the initial setting time decreases compared to the reference mortar, and when sand is substituted for RFCA, the time increases. In the first case, this is because of the addition of fine particles of hydrated cement to the mixture, which can act as crystallization nuclei and cause the products of this reaction to form in the inter-grain space [70]. The second case is different, and its explanation is based on the essence of the test method, and this may be an apparent increase. The test method consists of penetrating the mixture with a needle in a Vicat apparatus and determining the point at which it stops at the designated position. The idea behind the method is to maintain the same consistency of the mix. In the case of mortars with a standard aggregate, the aggregate does not affect the consistency of the mix and the ability of the Vicat apparatus needle to penetrate it. Such aggregate is hard, and upon contact with the needle, it will displace and create resistance to the needle’s movement. Lightweight aggregate is less mechanically resistant and can be penetrated by the Vicat apparatus needle and distort the test result.

It is difficult to compare exact values of compressive strength and thermal conductivity coefficient with results from the literature due to differences in the materials used and their proportions. The reported development of mechanical properties, density, and absorbability is similar to those reported in the literature for other lightweight aggregates [37,65,66,67,68,71]. In shaping the mechanical properties of composites containing lightweight aggregates, the effective w/c ratio has a strong influence [36,42]. This is a greater influence than for ordinary aggregates. This effect is observed in the present study. Many of the cited articles deal only with the mechanical properties of cementitious composites, without mentioning the thermal properties, which in the case of a material with insulating properties is very important. Below in Table 3 are shown the results of compressive strength, thermal conductivity coefficient, and the thermomechanical index calculated from them, depending on the level of aggregate replacement. It can be seen that with increasing amounts of lightweight demolition waste, the thermomechanical index decreases. Thanks to it and by determining a satisfactory level of compressive strength, it is possible to determine the optimal aggregate composition for new composites.

In the case of replacing cement with ground demolition waste originating from lightweight materials, there are far fewer studies. However, it can be seen that they can positively affect the compressive strength of cement mortars. This applies, however, to a limited amount of them. The results given in Table 4 are for AAC, which does not contain a foaming agent that can affect the mechanical properties of cementitious composites.

## 5. Conclusions

The research presented in this article was conducted to evaluate the suitability of specific demolition waste for reuse as an ingredient in cement mortars. The simultaneous determination of thermal and strength properties was performed. The aim of the research was to determine and compare the possibilities and amounts of waste used as a substitute for sand or cement.

1. Waste from the production of foam concrete can be used in the preparation of mortars. When added in small quantities, it causes an acceptable deterioration in mechanical properties while improving thermal properties. This material can be used especially in the production of plasters and mortars.
2. Replacing part of the sand with waste foam concrete improves the thermomechanical index, defined as the ratio between compressive strength and thermal conductivity coefficient. This shows the suitability of this material. Replacing part of the cement reduces the value of this index.
3. There is no need to over-mill RFCD. The results of the density, absorbability, and compressive strength tests show that replacing the cement with RFCD with a maximum grain size of 0.063 mm and 0.125 mm in the same amount does not affect these values.
4. The linear correlations of density—thermal conductivity coefficient and compressive strength—thermal conductivity coefficient are good for both the replacement of the cement part with RFCD and the sand part with RFCA.

## Figures and Tables

**Table 1:** Composition of mortars.

Symbol	Cement	NS	RFC (Max Grain (mm))			Water	SP	Water	SP
			0.063	0.125	4	For Initial Setting Time Tests ^1^		For Consistency Tests and Forming of Samples ^2^	
	(g)	(g)	(g)	(g)	(g)	(g)	(g)	(g)	(g)
REF	450	1350				225	0.91	225	0.35
C25	337.5	1350		112.5		225	4.50	225	2.9
C25′	337.5	1350	112.5			225	4.65	225	3.0
C50	225	1350		225		X ^3^		225	12.0
P10	450	1215			135	225	18.0	225	5.55
P20	450	1080			270	X ^3^		225	22.5
REF (W+)	450	1350				225	0 ^4^	225	0 ^5^
P10 (W+)	450	1215			135	225	8.5	225	3.15
P20 (W+)	450	1080			270	225	15.2	225	8.15
P30 (W+)	450	945			405	X ^3^		225	22.5

^1^ According to EN 196-3 [57], the water amount is changed. In current study, standard consistency is obtained with increasing superplasticizer content. ^2^ According to EN 196-1 [58] and EN 1015-3 [59]. ^3^ Not possible to obtain standard consistency (acc. to EN 196-3 [57]) with max allowed admixture dosage (5% of cement mass). ^4^ Consistency was slightly more liquid than EN 196-3 [57] standard requirements (plunger stopped 1 mm over base plate (3–9 mm required). ^5^ Consistency was more liquid than for other mortars from (W+) series.

**Table 2:** Properties of natural sand and RFCA.

	Bulk Density (Loose)	Fractions (mm)						
		0.0–0.063	0.063–0.125	0.125–0.25	0.25–0.5	0.5–1.0	1.0–2.0	2.0–4.0
	(g/dm^3^)	(%)						
NS	1627	0.5	1.4	9.7	41.1	43.6	3.7	
RFCD (max 0.063 mm)	550	100						
RFCD (max 0.125 mm)	480	45	55					
RFCA (max 4 mm)	412	6.1	8.3	12.4	17.9	27.5	22.3	5.5

**Table 3:** Compressive strength and thermal conductivity coefficient of composites containing lightweight concrete in crushed form as a substitute for aggregate after 28 days, according to [48,49,51,68].

Reference	Aggregate Used	Replacement Level	Compressive Strength (MPa)	Thermal Conductivity Coefficient (W/mK)	Thermomechanical Index Acc. to [64]
[48]	Crushed cellular concrete	100% natural aggregate	33.2	2.07	16.04
		20% recycled coarse aggregate	22.4	1.96	11.43
		40% recycled coarse aggregate	12.33	1.74	7.09
		60% recycled coarse aggregate	13.47	1.63	8.26
		100% recycled coarse aggregate	9.05	1.32	6.86
		100% recycled coarse aggregate; 100% recycled fine aggregate	8.40	1.18	7.12
[49]	AAC	100% natural sand	24	n/d	
		15% crushed AAC as fine aggregate	20		
		30% crushed AAC as fine aggregate	12		
	CLC	100% natural sand	24		
		15% crushed CLC as fine aggregate	19		
		30% crushed CLC as fine aggregate	9		
[51]	ACC	100% recycled aggregate *	12–16	0.39–0.45	30.77–35.56
		100% recycled aggregate *	6–9	0.28–0.36	21.43–25.00
		100% recycled aggregate *	2.5	0.28–0.36	8.93–6.94
[72]	ACC	100% natural aggregate	33.8	n/d	
		10% recycled AAC 0.15–4.75 mm	33.8		
		20% recycled AAC 0.15–4.75 mm	32.6		
		50% recycled AAC 0.15–4.75 mm	26.9		
		10% recycled AAC 0.15–0.3 mm	35.8		
		20% recycled AAC 0.15–0.3 mm	38.1		
		50% recycled AAC 0.15–0.3 mm	37.2		
		10% recycled AAC 0.3–4.75 mm	33.2		
		20% recycled AAC 0.3–4.75 mm	31.9		
		50% recycled AAC 0.3–4.75 mm	25.7		

* Various mix composition (cement, w/c ratio, etc.). n/d means “no data”.

**Table 4:** Compressive strength of composites containing lightweight concrete in ground form as a substitute for cement after 28 days, according to [49].

Reference	Material Used	Replacement Level	Compressive Strength (MPa)
[49]	Ground AAC	100% cement	24
		15% ground AAC	22
		30% ground AAC	18
	Ground CLC	100% cement	24
		15% ground CLC	27
		30% ground CLC	24

## Data Availability

Results are available on request.

## References

[1] 2022 Global Status Report for Buildings and Construction|UNEP—UN Environment Programme. https://www.unep.org/resources/publication/2022-global-status-report-buildings-and-construction.

[2] U.S. Geological Survey Mineral Commodity Summaries 2022—Cement. https://pubs.usgs.gov/periodicals/mcs2022/mcs2022-cement.pdf.

[3] Lehne J., Preston F. (2018). Making Concrete Change: Innovation in Low-Carbon Cement and Concret.

[4] Lehner P., Horňáková M., Pizoń J., Gołaszewski J. (2022). Effect of Chemical Admixtures on Mechanical and Degradation Properties of Metallurgical Sludge Waste Concrete. Materials.

[5] Alwaeli M., Gołaszewski J., Niesler M., Pizoń J., Gołaszewska M. (2020). Recycle Option for Metallurgical Sludge Waste as a Partial Replacement for Natural Sand in Mortars Containing CSA Cement to Save the Environment and Natural Resources. J. Hazard. Mater..

[6] Pizoń J., Gołaszewski J., Alwaeli M., Szwan P. (2020). Properties of Concrete with Recycled Concrete Aggregate Containing Metallurgical Sludge Waste. Materials.

[7] Poranek N., Łaźniewska-Piekarczyk B., Czajkowski A., Pikoń K. (2021). Circular Economy for Municipal Solid Waste Incineration Bottom Ash (MSWIBA) Management in Mortars with CSA and CEM I, MSWIBA Glassy Phase, and DTG. Energies.

[8] Poranek N., Łaźniewska-Piekarczyk B., Lombardi L., Czajkowski A., Bogacka M., Pikoń K. (2022). Green Deal and Circular Economy of Bottom Ash Waste Management in Building Industry—Alkali (NaOH) Pre-Treatment. Materials.

[9] Zhao T., Lv Y., Chen J., Song P., Sun M., Zhang X., Huang L. (2023). Effect of Glass Fiber-Reinforced Plastic Waste on the Mechanical Properties of Concrete and Evaluation of Its Feasibility for Reuse in Concrete Applications. Materials.

[10] Utilizing A., Chen Z., Hua J., Wang N., Kamal Askar M., S Al-Kamaki Y.S., Hassan A. (2023). Utilizing Polyethylene Terephthalate PET in Concrete: A Review. Polymers.

[11] Helmy S.H., Tahwia A.M., Mahdy M.G., Abd Elrahman M., Abed M.A., Youssf O. (2023). The Use of Recycled Tire Rubber, Crushed Glass, and Crushed Clay Brick in Lightweight Concrete Production: A Review. Sustainability.

[12] Mahat M., Acharya M., Acharya M., Mashal M. (2023). Use of Waste Tires as Transverse Reinforcement and External Confinement in Concrete Columns Subjected to Axial Loads. Sustainability.

[13] Green E., Jaya P., Sheikh Hassani M., Matos J.C., Zhang Y., Teixeira E.R. (2023). Green Concrete with Glass Powder—A Literature Review. Sustainability.

[14] Epure C., Munteanu C., Istrate B., Harja M., Buium F. (2023). Applications of Recycled and Crushed Glass (RCG) as a Substitute for Natural Materials in Various Fields—A Review. Materials.

[15] Cerchione R., Colangelo F., Farina I., Ghisellini P., Passaro R., Ulgiati S. (2023). Life Cycle Assessment of Concrete Production within a Circular Economy Perspective. Sustainability.

[16] Hubert J., Zhao Z., Michel F., Courard L. (2023). Effect of Crushing Method on the Properties of Produced Recycled Concrete Aggregates. Buildings.

[17] Hamada H.M., Shi J., Abed F., Al Jawahery M.S., Majdi A., Yousif S.T. (2023). Recycling Solid Waste to Produce Eco-Friendly Ultra-High Performance Concrete: A Review of Durability, Microstructure and Environment Characteristics. Sci. Total Environ..

[18] Chaudhary N., Mohanty I., Saha P., Kumari R., Kumar Pandey A. (2023). Performance of Resource Saving Agro-Industrial Wastes and Their Utilization in Lightweight Concrete—A Review. Mater. Today Proc..

[19] Jahami A., Issa C.A. (2023). Exploring the Use of Mixed Waste Materials (MWM) in Concrete for Sustainable Construction: A Review. Constr. Build. Mater..

[20] Raj A., Sathyan D., Mini K.M. (2019). Physical and Functional Characteristics of Foam Concrete: A Review. Constr. Build. Mater..

[21] Xiao J., Hao L., Cao W., Ye T. (2022). Influence of Recycled Powder Derived from Waste Concrete on Mechanical and Thermal Properties of Foam Concrete. J. Build. Eng..

[22] Gołaszewski J., Klemczak B., Smolana A., Gołaszewska M., Cygan G., Mankel C., Peralta I., Röser F., Koenders E.A.B. (2022). Effect of Foaming Agent, Binder and Density on the Compressive Strength and Thermal Conductivity of Ultra-Light Foam Concrete. Buildings.

[23] Othuman M.A., Wang Y.C. (2011). Elevated-Temperature Thermal Properties of Lightweight Foamed Concrete. Constr. Build. Mater..

[24] Zhao W., Su Q., Wang W., Niu L., Liu T. (2018). Experimental Study on the Effect of Water on the Properties of Cast in Situ Foamed Concrete. Adv. Mater. Sci. Eng..

[25] Jones M.R., McCarthy A. (2015). Behaviour and Assessment of Foamed Concrete for Construction Applications. Use of Foamed Concrete in Construction.

[26] Shang X., Qu N., Li J. (2022). Development and Functional Characteristics of Novel Foam Concrete. Constr. Build. Mater..

[27] Mydin M.A.O., Wang Y.C. (2012). Mechanical Properties of Foamed Concrete Exposed to High Temperatures. Constr. Build. Mater..

[28] Jitchaiyaphum K., Sinsiri T., Chindaprasirt P. (2011). Cellular Lightweight Concrete Containing Pozzolan Materials. Procedia Eng..

[29] Tan X., Chen W., Wang J., Yang D., Qi X., Ma Y., Wang X., Ma S., Li C. (2017). Influence of High Temperature on the Residual Physical and Mechanical Properties of Foamed Concrete. Constr. Build. Mater..

[30] Irawan T., Saloma, Idris Y. (2019). Mechanical Properties of Foamed Concrete with Additional Pineapple Fiber and Polypropylene Fiber. J. Phys. Conf. Ser..

[31] Kozłowski M., Kadela M. (2018). Mechanical Characterization of Lightweight Foamed Concrete. Adv. Mater. Sci. Eng..

[32] Bakhshi M., Dalalbashi A., Soheili H. (2023). Energy Dissipation Capacity of an Optimized Structural Lightweight Perlite Concrete. Constr. Build. Mater..

[33] Topçu I.B., Işikdaǧ B. (2008). Effect of Expanded Perlite Aggregate on the Properties of Lightweight Concrete. J. Mater. Process. Technol..

[34] Al-Tarbi S.M., Baghabra Al-Amoudi O.S., Al-Osta M.A., Al-Awsh W.A., Shameem M., Sharif Zami M. (2023). Development of Energy-Efficient Hollow Concrete Blocks Using Perlite, Vermiculite, Volcanic Scoria, and Expanded Polystyrene. Constr. Build. Mater..

[35] Schackow A., Effting C., Folgueras M.V., Güths S., Mendes G.A. (2014). Mechanical and Thermal Properties of Lightweight Concretes with Vermiculite and EPS Using Air-Entraining Agent. Constr. Build. Mater..

[36] Ahmadi S.F., Reisi M., Sajadi S.M. (2023). Comparing Properties of Foamed Concrete and Lightweight Expanded Clay Aggregate Concrete at the Same Densities. Case Stud. Constr. Mater..

[37] Bideci A., Bideci Ö.S., Ashour A. (2023). Mechanical and Thermal Properties of Lightweight Concrete Produced with Polyester-Coated Pumice Aggregate. Constr. Build. Mater..

[38] Muhtar (2023). Performance-Based Experimental Study into Quality Zones of Lightweight Concrete Using Pumice Aggregates. Case Stud. Constr. Mater..

[39] Graziano S.F., Zanelli C., Molinari C., de Gennaro B., Giovinco G., Correggia C., Cappelletti P., Dondi M. (2022). Use of Screen Glass and Polishing Sludge in Waste-Based Expanded Aggregates for Resource-Saving Lightweight Concrete. J. Clean. Prod..

[40] Liang Y., Wang Q., Gan W., Liao J., Lai M., Ho J. (2022). A 14-Year Study on Ceramic Waste Slag-Based Lightweight Aggregate Concrete. Constr. Build. Mater..

[41] Muthusamy K., Budiea AM A., Zaidan AL F., Rasid M.H., Hazimmah D.S. (2017). Properties of Concrete Containing Foamed Concrete Block Waste as Fine Aggregate Replacement. IOP Conf. Ser. Mater. Sci. Eng..

[42] Domagała L. (2015). The Effect of Lightweight Aggregate Water Absorption on the Reduction of Water-Cement Ratio in Fresh Concrete. Procedia Eng..

[43] Castro J., Keiser L., Golias M., Weiss J. (2011). Absorption and Desorption Properties of Fine Lightweight Aggregate for Application to Internally Cured Concrete Mixtures. Cem. Concr. Compos..

[44] Kim Y.H., Park C.B., Choi B.I., Shin T.Y., Jun Y., Kim J.H. (2020). Quantitative Measurement of Water Absorption of Coarse Lightweight Aggregates in Freshly-Mixed Concrete. Int. J. Concr. Struct. Mater..

[45] Petrella A., Di Mundo R., Notarnicola M. (2020). Recycled Expanded Polystyrene as Lightweight Aggregate for Environmentally Sustainable Cement Conglomerates. Materials.

[46] Ben Fraj A., Kismi M., Mounanga P. (2010). Valorization of Coarse Rigid Polyurethane Foam Waste in Lightweight Aggregate Concrete. Constr. Build. Mater..

[47] Wang J., Zheng K., Cui N., Cheng X., Ren K., Hou P., Feng L., Zhou Z., Xie N. (2020). Green and Durable Lightweight Aggregate Concrete: The Role of Waste and Recycled Materials. Materials.

[48] Borhan T.M. (2015). Effect of Using Recycled Lightweight Aggregate on the Properties of Concrete. J. Univ. Babylon.

[49] Lalrinmawii E., Sahu S., Sarkar P., Davis R. (2020). Feasible Use of Recycled Foam Concrete in Cement Mortar. IOP Conf. Ser. Mater. Sci. Eng..

[50] Gyurkó Z., Jankus B., Fenyvesi O., Nemes R. (2019). Sustainable Applications for Utilization the Construction Waste of Aerated Concrete. J. Clean Prod..

[51] Fenyvesi O., Jankus B. (2015). Opportunities in Recycling AAC Waste as Aggregate for Lightweight Concrete. Epa. J. Silic. Based Compos. Mater..

[52] Land G., Stephan D. (2018). The Effect of Synthesis Conditions on the Efficiency of C-S-H Seeds to Accelerate Cement Hydration. Cem. Concr. Compos..

[53] Liu M., Hu R., Zhang Y., Wang C., Ma Z. (2023). Effect of Ground Concrete Waste as Green Binder on the Micro-Macro Properties of Eco-Friendly Metakaolin-Based Geopolymer Mortar. J. Build. Eng..

[54] Bouarroudj M.E.K., Rémond S., Bulteel D., Potier G., Michel F., Zhao Z., Courard L. (2021). Use of Grinded Hardened Cement Pastes as Mineral Addition for Mortars. J. Build. Eng..

[55] Ma X., Wang Z. (2013). Effect of Ground Waste Concrete Powder on Cement Properties. Adv. Mater. Sci. Eng..

[56] Recycling Old A., Li L., Zhang S., Li X., Xiao J., Zhu Xie H., Gu Li L., Liu F., Kwok Hung Kwan A. (2022). Recycling Old Concrete as Waste Concrete Powder for Use in Pervious Concrete: Effects on Permeability, Strength and Eco-Friendliness. Buildings.

[57] (2016). Methods of Testing Cement—Part 3: Determination of Setting Times and Soundness.

[58] (2018). Methods of Testing Cement—Part 1: Determination of Strength.

[59] (2007). Methods of Test for Mortar for Masonry—Part 3: Determination of Consistence of Fresh Mortar (by Flow Table).

[60] (2007). Admixtures for Concrete, Mortar, and Grout—Test Methods—Part 2: Determination of Setting Time.

[61] (2007). Methods of Test for Mortar for Masonry—Part 6: Determination of Bulk Density of Fresh Mortar.

[62] (2007). Methods of Test for Mortar for Masonry—Part 10: Determination of Dry Bulk Density.

[63] Pizon J., Lazniewska-Piekarczyk B. (2019). Comparison of Efficiency of Accelerating Admixtures for Concrete Using Multiple-Criteria Decision Analysis (MCDA). IOP Conf. Ser. Mater. Sci. Eng..

[64] Nowak J., Jaskulski R., Kubissa W., Matusiak B., Banach M. (2023). On the Need for a Paradigm Change in the Valuation of Concrete with Waste Materials Based on the Example of Concrete with Crumb Rubber. Sustainability.

[65] Ding Y., She A., Yao W. (2023). Investigation of Water Absorption Behavior of Recycled Aggregates and Its Effect on Concrete Strength. Materials.

[66] García-González J., Rodríguez-Robles D., Juan-Valdés A., Pozo J.M.M.d., Guerra-Romero M.I. (2014). Pre-Saturation Technique of the Recycled Aggregates: Solution to the Water Absorption Drawback in the Recycled Concrete Manufacture. Materials.

[67] Domagała L., Bryła E., Oh J.-E., Yoon S.-Y. (2021). The Properties of Lightweight Aggregates Pre-Coated with Cement Pastes and Their Suitability for Concrete. Materials.

[68] Lo T.Y., Cui H.Z. (2004). Effect of Porous Lightweight Aggregate on Strength of Concrete. Mater. Lett..

[69] Łaźniewska-Piekarczyk B. (2020). The Influence of Superplasticizer and Shrinkage Reducing Admixture Type on Air-Content and Related Properties of HPSCC. MATEC Web. Conf..

[70] Wang B., Yao W., Stephan D. (2019). Preparation of Calcium Silicate Hydrate Seeds by Means of Mechanochemical Method and Its Effect on the Early Hydration of Cement. Adv. Mech. Eng..

[71] Wichmann I., Stephan D. (2023). Mechanical and Physical Properties of Concrete Made of Alkali-Activated Lightweight Aggregates from Construction Demolition Waste. Mater Today Proc..

[72] Zou D., Que Z., Cui W., Wang X., Guo Y., Zhang S. (2022). Feasibility of Recycling Autoclaved Aerated Concrete Waste for Partial Sand Replacement in Mortar. J. Build. Eng..

